# Knee4Life: Empowering Knee Recovery After Total Knee Replacement Through Digital Health Protocol

**DOI:** 10.3390/s24227334

**Published:** 2024-11-17

**Authors:** Maedeh Mansoubi, Phaedra Leveridge, Matthew Smith, Amelia Fox, Garry Massey, Sarah E. Lamb, David J. Keene, Paul Newell, Elizabeth Jacobs, Nicholas S. Kalson, Athia Haron, Helen Dawes

**Affiliations:** 1 Medical School, University of Exeter, Exeter EX1 2LU, UK; m.mansoubi@exeter.ac.uk (M.M.); ms893@exeter.ac.uk (M.S.); ameliafox404@gmail.com (A.F.); g.j.massey@exeter.ac.uk (G.M.); s.e.lamb@exeter.ac.uk (S.E.L.); d.keene@exeter.ac.uk (D.J.K.); p.newell@exeter.ac.uk (P.N.); h.dawes@exeter.ac.uk (H.D.); 2 NIHR Exeter Biomedical Research Centre, Medical School, Faculty of Health and Life Sciences, University of Exeter, Exeter EX1 2LU, UK; 3 Intersect@Exeter, Medical School, University of Exeter, Exeter EX1 2LU, UK; 4 Royal Devon and Exeter NHS Foundation Trust, Barrack Road, Exeter EX2 5DW, UK; liz.jacobs@nhs.net; 5 Northumbria Healthcare NHS Foundation Trust, Queen Victoria Rd, Newcastle upon Tyne NE1 4LP, UK; nicholas.kalson@nhs.net; 6 Department of Mechanical, Aerospace and Civil Engineering (MACE), University of Manchester, Manchester M13 9PL, UK; athia.haron@manchester.ac.uk

**Keywords:** computer vision, digital health, knee stiffness, remote patient monitoring, total knee replacement

## Abstract

Pain and knee stiffness are common problems following total knee replacement surgery, with 10–20% of patients reporting dissatisfaction following their procedure. A remote assessment of knee stiffness could improve outcomes through continuous monitoring, facilitating timely intervention. Using machine learning algorithms, computer vision can extract joint angles from video footage, offering a method to monitor knee range of motion in patients’ homes. This study outlines a protocol to provide proof of concept and validate a computer vision-based approach for measuring knee range of motion in individuals who have undergone total knee replacement. The study also explores the feasibility of integrating this technology into clinical practice, enhancing post-operative care. The study is divided into three components: carrying out focus groups, validating the computer vision-based software, and home testing. The focus groups will involve five people who underwent total knee replacement and ten healthcare professionals or carers who will discuss the deployment of the software in clinical settings. For the validation phase, 60 participants, including 30 patients who underwent total knee replacement surgery five to nine weeks prior and 30 healthy controls, will be recruited. The participants will perform five tasks, including the sit-to-stand test, where knee range of motion will be measured using computer vision-based markerless motion capture software, marker-based motion capture, and physiotherapy assessments. The accuracy and reliability of the software will be evaluated against these established methods. Participants will perform the sit-to-stand task at home. This will allow for a comparison between home-recorded and lab-based data. The findings from this study have the potential to significantly enhance the monitoring of knee stiffness following total knee replacement. By providing accurate, remote measurements and enabling the early detection of issues, this technology could facilitate timely referrals to non-surgical treatments, ultimately reducing the need for costly and invasive procedures to improve knee range of motion.

## 1. Introduction

Total knee replacement (TKR) is a common procedure, with over 100,000 operations performed annually in England and Wales [1]. While most patients experience successful outcomes following TKR, approximately 10–20% of patients are dissatisfied—primarily due to pain and knee stiffness [2]. A recent James Lind Alliance Priority Setting Partnership identified ways to understand the causes of knee stiffness after TKR, how it can be prevented, and the best treatments as a top-10 priority for clinical research [3]. A method to detect early problems with pain and stiffness could facilitate earlier referral to non-surgical treatments which are effective in preventing the need for more invasive treatments, such as manipulation under anaesthetic [4].

Our current understanding of the onset, timing, and optimal treatment for stiffness is limited. An accurate remote assessment of knee stiffness could improve outcomes through continuous monitoring and timely interventions. This will reduce the need for frequent clinic visits. Knee stiffness post-TKR is defined as range of motion (ROM) limitation and is often associated with pain [5,6]. ROM is typically measured using a handheld goniometer or through a visual assessment by a clinician. Both methods have been shown to be inaccurate [7]; therefore, a more precise method of measurement is required. Furthermore, these methods require trained healthcare staff, so they are impractical for remote assessment. Therefore, the ideal tool for measuring knee stiffness should be accurate, easy to use, and capable of providing rapid feedback to both patients and clinical teams.

Markerless pose estimation using computer vision has the potential to be used by patients to improve remote rehabilitation [8]. Computer vision is a branch of artificial intelligence that uses machine learning algorithms to automate the analysis of human movement from videos. A computer vision-based approach has been validated for the clinical assessment of spine mobility in patients with ankylosing spondylitis [9]. However, it is important to validate machine learning algorithms for the TKR population to account for individual differences in movement patterns. By providing accurate knee flexion measurements, computer vision could enhance the assessment of knee ROM and facilitate targeted interventions. This study aims to develop and validate a computer vision-based approach to monitor knee flexion and extension.

Functional tasks that reflect daily movements, such as the sit-to-stand task, could be used to monitor knee ROM. These tasks are easier to complete in the home environment than goniometer measurements and represent functional ability. The sit-to-stand test is recommended for monitoring recovery from TKR surgery [10]. Evidence suggests that sagittal plane knee ROM is decreased during the sit-to-stand task after TKR surgery compared to healthy controls [11], indicating that this task may be used to measure ROM in post-operative patients. Therefore, this study will use the sit-to-stand test to measure ROM in a home-based setting.

The computer vision-based approach offers a non-invasive, objective method to monitor knee flexion in patients with knee stiffness, which could inform treatment decisions to improve outcomes. Currently, no tool exists to remotely and accurately detect early post-surgical knee stiffness. This study seeks to develop a cost-effective tool for measuring and quantifying knee stiffness pre- and post-TKR surgery for use across the NHS. This study also aims to determine its reliability, feasibility, and usability for implementation in the home.

The primary objectives of the study are the following:

1. To provide proof of concept for a computer vision-based approach that measures knee ROM for use in the home setting.
2. To validate the computer vision-based approach in people who have undergone TKR by ascertaining the test–retest reliability of algorithm outputs such as knee flexion/extension angles.
3. To investigate the integration of the computer vision-based approach into clinical practice through focus groups and identify key development areas for successful implementation.

The secondary objectives of the study are the following:

1. To explore how pain influences knee ROM in people who undergone TKR.
2. To investigate the relationship between muscle activation and ROM in people who have undergone TKR and in healthy controls.
3. To describe the association between physical activity and ROM in people who have undergone TKR.
4. To identify the potential risk factors, measured using questionnaires, linked with knee stiffness post-TKR surgery.

This study will be split into three stages: (1) focus groups, (2) validation of the computer vision-based approach for measuring knee range of motion in the laboratory, and (3) home testing. These will be outlined and addressed separately within the protocol.

## 2. Experimental Design

This project will be underpinned by the new Medical Research Council guidelines for developing a complex intervention [12]. It will use a participatory design methodology that uses evidence-based research and behaviour change models to identify intrinsic and extrinsic factors that contribute to a given outcome in a specific population.

### 2.1. Focus Groups

Focus groups will be conducted with people who have undergone TKR surgery (*n* = 5) and healthcare professionals and carers (*n* = 10). Discussions will provide valuable insights into the future adoption and application of the computer vision-based approach.

Staff involved in rehabilitation or care provision (clinicians, carers, and managers; *n* = 10) will be convened to discuss how the computer vision-based approach will be deployed. We will identify barriers and facilitators to deployment and report how these should be addressed.

People who have undergone TKR surgery (*n* = 5) will explore potential issues that may be experienced when using the system in their homes. We will explore support structures (e.g., social networks) and barriers/facilitators to engagement. Potential software, hardware, support, and training changes will be mapped and reported. Patient/carer instruction materials will be created.

A final service and product specification, including reference to the materials above, will be prepared to inform the second stage of this study.

#### 2.1.1. Inclusion and Exclusion Criteria

The inclusion criteria are as follows:

- People aged ≥18 years old;
- People who have undergone total knee replacement surgery or those who have worked with people following this surgery as a clinician, carer, or therapist;
- Individuals who are able to give informed consent;
- Individuals who are able to communicate in English with the research team.

The exclusion criteria are as follows:

- Individuals with any medical condition compromising their safety or ability to take part in the study;
- Individuals unable to adhere to the study procedures.

#### 2.1.2. Sampling Technique

Convenience sampling will be used to recruit healthcare professionals/carers and people who have undergone TKR surgery. Convenience sampling is appropriate because the research is exploratory in nature. The research team will identify them through professional networks and associations to invite them to take part in the study. Participants who underwent TKR surgery will be identified through participant engagement groups.

### 2.2. Validation of Computer Vision-Based Approach

#### 2.2.1. Study Setting

This study will be conducted at the University of Exeter’s Richards Building, St Luke’s Campus. Participants will be recruited from the Princess Elizabeth Orthopaedic Centre based at the Royal Devon and Exeter, the Nightingale Hospital Exeter, and the North Devon District Hospital.

#### 2.2.2. Sample Size

Sixty participants (thirty participants who underwent total knee surgery five to nine weeks prior and thirty healthy controls) will be recruited for the project. Formal sample size estimates are not required for pilot and feasibility studies (PAFs). The choice of sample size (60 participants, 40 with complete data) reflects a balance between a large enough sample to represent population diversity and provide estimates of variability while ensuring that recruitment is achievable (participant drop-out and approximately 50% of eligible participants recruited). While there are no specific recommendations for sample sizes for PAFS, a general rule of thumb suggests a minimum of 30 people [13]. Twenty to thirty people are generally sufficient to estimate the standard deviation of outcome measures to estimate the sample size for a definitive clinical trial based on known estimates of Minimal Clinically Important Differences [14].

For the chosen sample size of 30 participants in each group, the power to detect varying effect sizes at a statistical significance level of 5% is shown in Table 1.

#### 2.2.3. Sampling Technique

Convenience sampling will be used to recruit healthy adults for this study. A purposive sampling method will also be used, where participants who underwent TKR will be identified through hospital records and invited to participate by the NHS trust research team. Participants who underwent TKR will be recruited from the Princess Elizabeth Orthopaedic Centre based at the Royal Devon and Exeter and the Nightingale Hospital Exeter. Convenience sampling and purposive sampling are appropriate because this research is exploratory in nature.

#### 2.2.4. Inclusion and Exclusion Criteria

Inclusion Criteria—Group of Participants who underwent TKR:

- Adults (aged 18 years and over);
- Individuals who have undergone total knee replacement in the past five to nine weeks.

Inclusion Criteria—Healthy Adult Group:

- Adults (aged 18 years and over);
- Participants with no previous musculoskeletal injuries (past six months) or current neuro-musculoskeletal disorders that affect standing and walking.

Exclusion Criteria:

- Individuals with cognitive impairment affecting their ability to participate and follow instructions safely;
- Individuals with any skin conditions or broken skin in the calf and behind the knee area;
- Individuals with deep brain stimulation or pacemaker implants or other implants that may interfere with the measurement system.

### 2.3. Home Testing

Participants recruited for the computer vision-based approach validation will then undertake the home testing procedure. The home testing protocol will take place in the participants’ own homes.

## 3. Materials and Methods

### 3.1. Focus Groups

Interviews will be conducted in a hybrid setting and recorded via Microsoft (MS) Teams (Microsoft, Washington, DC, USA). Interview transcripts will be coded using NVivo 15 software (Lumivero, Denver, CO, USA).

### 3.2. Validation of Computer Vision-Based Approach

#### 3.2.1. Physical Activity Assessment

Daily physical activity and sedentary time will be assessed using the Axivity AX3, a ‘wrist-worn’ tri-axial accelerometer (Open Lab, Newcastle University, Newcastle upon Tyne, UK). AX3 data will be downloaded using OMGUI software (open movement (V.1.0.0.37) and analysed using DigiPA software (version 4.01).

#### 3.2.2. Questionnaires

Questionnaire data will be collected using REDCap electronic data capture tools available at the University of Exeter. REDCap (Research Electronic Data Capture) is a secure, web-based software platform designed to support data capture for research studies [15,16]. Participants will be asked to complete the following seven questionnaires:

1. A questionnaire on demographic data. This will include clinical scores and comorbidities (age, sex, ethnicity, handedness, smoking, marital status, drug history, and other medical conditions).
2. The PROMIS-29 v2 (Patient-Reported Outcomes Measurement Information System). This measures both physical and mental health through a concise set of questions [17].
3. The Oxford Knee Score (OKS). This is a validated measure used to assess pain and function in individuals who underwent total knee replacement or those who are suffering from knee arthritis [18].
4. The Numeric Rating Scale (NRS). This is a pain assessment tool where individuals rate their pain on a numeric scale, typically ranging from 0 to 10, with 0 being no pain and 10 indicating the worst possible pain.
5. The IPAQ Short Last 7 Days. This is the International Physical Activity Questionnaire used to assess physical activity in the past week. It measures moderate and vigorous activity along with walking in 10-min bouts within seven days [19].
6. The Barthel Index for Activities of Daily Living (ADL). This measures functional independence, especially in patients who experienced a stroke or those with chronic disabilities. This ordinal scale focuses on personal care and mobility [20].
7. The EQ-5D-5L. This is a health assessment tool that measures health status across five dimensions: mobility, self-care, usual activities, pain/discomfort, and anxiety/depression [21].

#### 3.2.3. Motion Capture

Each participant will be provided with a tight-fitting t-shirt and gym shorts. A full-body set of retroreflective markers (*n* = 60) based on the conventional gait model 2 [22] will be applied to the participant using surgical tape. Marker-based motion capture data will be collected using 12 Vicon Valkyrie VK16 cameras (Vicon, Oxford, UK). Data will be recorded at 100 Hz. Motion capture data will be processed in Vicon Nexus software (version 2.16) and analysed in Visual3D (C-Motion, Rockville, MD, USA).

Computer vision-based markerless motion capture will be run on two laptops to capture data: in the sagittal plane and at a 30° angle. Digimotion software (version 5.01) will be run locally on each laptop to activate the webcam and calculate hip, knee, and ankle angles in real time. Data will be recorded at 30 Hz. Two video cameras and three iPads/iPhones (Apple, Cupertino, CA, USA) will record videos from different angles to determine any angle effect. Digimove software (version 4.01) will calculate hip, knee and ankle angles from recorded videos. Videos will be recorded at 30 Hz.

#### 3.2.4. Surface Electromyography

Surface electromyography (sEMG) data will be recorded using Trigno Avanti Sensors (2148 Hz, Trigno Wireless System, Delsys, Sale, UK). Twelve sEMG electrodes will be placed bilaterally to avoid the general location of innervation zones and distal tendon areas [23] over the following muscles: vastus lateralis, vastus medialis, semitendinosus, biceps femoris, tibialis anterior, and the medial head of the gastrocnemius.

### 3.3. Home Testing

Participants will use their own mobile phones or tablet devices to record a video of themselves completing the movement tasks. Participants will upload their videos to REDCap [15,16].

## 4. Procedure Details

### 4.1. Focus Groups

Focus groups will be conducted in a hybrid environment, where participants can attend in person or via MS Teams. Initially, separate focus group sessions will be conducted with staff and service user groups. Printed question sheets will guide group discussion. Each group will have a researcher facilitating the discussion. Following the separate focus group sessions, groups will be mixed to enable a more diverse discussion perspective. Each focus group session will last 75–90 min.

#### Thematic Analysis

A thematic content analysis of the interview transcripts will be used to investigate the usability and perceived usefulness of the computer vision-based approach (by clinicians and people who underwent TKR). It will also be used to explore participants’ experiences of symptoms.

Focus group transcripts will be transcribed verbatim. Two reviewers will independently code each interview, and any discrepancies identified after the initial four transcripts will be resolved. An interim analysis of the first 15 participants will determine if thematic saturation has been achieved.

To explore usability factors, the transcripts will undergo a thematic analysis. Personal documentation will be initially reviewed through a content analysis, with a more detailed thematic approach applied if needed. Data inconsistencies will guide a member-checking process with participants, conducted before the write-up and dissemination of findings.

### 4.2. Validation of Computer Vision-Based Approach

Participants will wear an activity monitoring accelerometer for one week before the experimental session at the university. The accelerometer will be removed at the session. Physical activity monitoring can be repeated in the week following the visit if there are any problems with the data.

Motion capture markers and sEMG electrodes will then be applied. A physiotherapist will administer questionnaires.

A static trial will be recorded, followed by a series of gait/functional tasks. Participants will complete a two-minute walk on a treadmill at a self-selected walking speed. A sit-to-stand test will then be completed three times: participants will start in a seated position and stand up, then sit down without the use of their hands.

Participants will then be asked to complete the timed-up-and-go test (TUG) three times. The participant will start by sitting down in a chair and will stand up, walk forward three metres, turn around a cone, and then walk back to the chair before sitting down. Finally, an overground walking task will be completed three times: the participant will be asked to walk five metres, turn around a cone, and walk another five metres.

Knee range of motion will be measured by a trained physiotherapist. The maximum and minimum angles will be measured using a universal goniometer whilst lying on a physiotherapy couch and whilst seated on a chair.

Participants will be offered the chance to rest between procedures for as long as preferred. The Single Ease Question (SEQ) is a concise post-task questionnaire utilising a seven-point rating scale to gauge the perception of the difficulty or ease of a specific task. The SEQ will be completed after every task. Following the completion of testing, all equipment will be removed from the participant.

#### Data Analysis 

Descriptive and frequency statistics will be reported for demographic, usability, acceptability, and feasibility data, and missing data will be reported.

To validate the motion capture measurements of the computer vision-based motion capture software against those from the marker-based motion capture software, we will use Bland–Altman (Tukey mean-difference) plots to assess for systematic deviation and determine any necessary bias correction.

To determine the test–retest reliability of the motion capture knee ROM measurements and to identify potential risk factors associated with knee stiffness, we will apply mixed-effects linear models for knee ROM adjusted for the group (group that underwent TKR or control) and questionnaire scores, with random effects for each participant. Variance components of the mixed-effects model may be used to estimate reliability measures as follows:

Relative reliability will be estimated using the intra-class correlation (2) (ICC) of Two-Way Random Effects [24]. Interpretation of ICC values will be conducted according to published guidance: an ICC of less than 0.5 is considered poor, between 0.5 and 0.75 is moderate, between 0.75 and 0.90 is good, and above 0.90 is excellent [25]. Absolute reliability will be estimated by the standard error of measurement (SEM) [26], calculated as $SD\left(standard\;deviation\right)\times \surd (1-ICC)$. The Minimal Detectable Change at the 95% confidence level (${MDC}_{95}$) is the minimal amount of change that would be accepted as a real change greater than that seen due to random variation or error [26]. MDC_95_ is calculated as follows: ${MDC}_{95}=1.96\times SEM\times \sqrt{2}$.

To explore the relationship between knee ROM and pain, muscle activation, physical activity, and potential risk factors, appropriate correlations/statistical tests will be selected to describe associations.

### 4.3. Home Testing

Participants will be asked to set up a camera device (mobile phone/tablet) in their homes and record themselves completing the following:

- The sit-to-stand test;
- If possible (within the limitations of the participants’ available space and ability to capture the trial), the TUG walk.

The research team will send the participants an email that will allow them to upload their video files to REDCAP. Videos will be analysed using a custom computer vision-based application. Participants will be asked to complete a System Usability Scale questionnaire [27,28]. The participants will be asked to complete this task within a week of the lab visit.

Descriptive and frequency statistics will be reported for the System Usability Scale questionnaire, and missing data will be reported. Knee ROM will be compared to the $MDC_{95}$ to determine compatibility with lab-based measurements.

## 5. Expected Results

This study aims to validate and investigate how a computer vision-based approach measuring knee ROM in people who have had TKR surgery can be used in clinical practice. The findings of this research project could lead to improvements in the diagnosis, monitoring, and management of knee stiffness after TKR. Unlike current methods—which require in-person evaluations and subjective assessments [7]—data could be captured over regular time points, providing a comprehensive view of the recovery trajectory.

Understanding the priority of patients and healthcare professionals from focus groups will enable consideration of how the computer vision-based approach can be scaled for use in healthcare settings. Consideration should be given to fitting the approach with regular clinical workflows and integrating it with existing healthcare records [29]. The barriers and facilitators to implementation will be identified—such as training requirements, infrastructure limitations, and user acceptance. Scaling the approach for use in broader healthcare settings will require addressing potential infrastructure challenges—including smartphone access and patient support—and data privacy considerations to protect sensitive videos [30]. The computer vision-based software can analyse videos in real time, meaning the videos do not need to be stored, thus minimising privacy risks and data storage demands.

Once the computer vision-based approach has been validated, adaptive machine learning models could support its implementation into the healthcare system. These models could adjust to the recovery trajectories of patients who underwent TKR and improve the timing of interventions. The establishment of personalised thresholds for interventions by using collected data to automate the system [31] could enable the early identification of pain and stiffness. This development will be guided by the results and insights gained from the focus groups to ensure alignment with user requirements.

The primary focus of this study is on short-term recovery from TKR surgery; however, the computer vision-based approach could also be used for longer-term monitoring. Knee ROM and peak flexion during gait are reduced in people with severe knee osteoarthritis compared to those with moderate or asymptomatic knee osteoarthritis [32]. Furthermore, peak knee flexion during stance progressively reduced between asymptomatic, moderate, and severe osteoarthritis groups [32]. This suggests that monitoring knee ROM—or other kinematic variables—could help identify early signs of osteoarthritis progression. Personalised thresholds could then be used to identify candidates for intervention, such as additional physiotherapy.

### 5.1. Strengths

The computer vision-based application only requires a single camera, such as a camera on a smartphone. Mobile phone literacy levels are high and are well accepted in research by older adults [33]. This accessibility makes the technology promising for widespread use in remote monitoring. As a result, it could lead to improved patient engagement and more frequent monitoring.

The computer vision-based software could help widen accessibility to specialists who may not be local. This could help provide better service access, particularly in more remote areas. This technology can be used alongside face-to-face telemedicine to improve remote patient care. Remote assessments are also valuable for monitoring and tracking changes in physical movements at increments unrealistic for in-person visits in the NHS. This could allow for the earlier identification of problems of stiffness, which would facilitate earlier intervention.

This study will also employ focus groups which can generate rich, qualitative data through interactive discussions. Comparisons made between participant experiences can provide valuable insights which would be lost in individual interviews [34]. Focus group discussions are flexible and allow for further insights into unexpected but relevant ideas to be obtained [35]. This will help provide a detailed understanding of the benefits and challenges of implementation into clinical practice.

### 5.2. Limitations

The computer vision-based approach is designed to assess only physical movements, so it cannot replace the requirement for clinician interaction. The software does not address wound care and symptoms beyond pain and swelling, so in these instances, input from healthcare professionals is required. The software should therefore be considered as an adjunct to current clinical practice rather than a replacement of appointments.

This study employs convenience sampling and purposive sampling methods. While these approaches are justified given the exploratory nature of the research, they may introduce selection bias, limiting the generalisability of the findings. To mitigate this, demographic questions will be asked, ensuring that the results are interpreted with context. Further research should employ more diverse sampling methods and larger sample sizes in randomised controlled trials to establish the utility of the computer vision-based approach in a clinical environment.

Focus groups tend towards consensus and may mask dissenting views [36]. Facilitators will build rapport with individuals before commencing the focus group and encourage group interaction to ensure participants feel comfortable sharing their views. Focus groups also tend to attract more confident individuals, which may result in the population being misinterpreted [37]. Healthcare professionals will be recruited from a wide range of clinical backgrounds to ensure varied opinions. Participants who underwent TKR will have a variety of experiences, which is unlikely to be linked with confidence levels.

### 5.3. Ethics and Dissemination

The protocol was evaluated and approved by the London—Chelsea Research Ethics Committee (IRAS ID 339937, REC reference 24/PR/0438) and will be carried out in accordance with the Declaration of Helsinki [38], the UK Policy Framework for Health and Social Care Research [39], and the general principles of Good Clinical Practice E6 (R2). Participant data will be pseudonymised and stored securely on the University of Exeter server.

Before any study-related procedure, the participant must sign a consent form, which states that they can withdraw at any time, for any reason, without affecting future care or needing to provide a reason. If a participant withdraws, their previously collected data will still be used. If the participant withdraws due to a serious adverse event, their GP or managing clinician will be followed up with as needed. This will be detailed in the Participant Information Sheet and consent form.

The findings will be disseminated through manuscript publications in peer-reviewed journals and conference presentations.

## 6. Conclusions

Computer vision-based markerless motion capture software could represent an accurate, easy-to-use method of measuring knee ROM, which can provide rapid feedback to patients and clinical teams. Our study will determine the reliability, feasibility, and usability of this approach for measuring knee ROM at home in people who have undergone TKR surgery. Focus groups will provide insights into the integration of the computer vision-based approach into clinical practice. The potential validation of the approach offers clinicians a tool to track recovery trajectories and identify issues earlier in the rehabilitation process.

## Figures and Tables

**Table 1:** Power to detect effect sizes at significance level of α = 0.05.

Effect size	0.2	0.3	0.4	0.5	0.6	0.7	0.8	0.9	1.0
Power	0.12	0.21	0.33	0.48	0.63	0.76	0.86	0.93	0.97

## Data Availability

Not applicable.
